# Survival of hematological patients after discharge from the intensive care unit: a prospective observational study

**DOI:** 10.1186/cc13172

**Published:** 2013-12-30

**Authors:** Teresa Bernal, Estefanía V Pardavila, Juan Bonastre, Isidro Jarque, Marcio Borges, Joan Bargay, Jose Ignacio Ayestarán, Josu Insausti, Pilar Marcos, Victor González-Sanz, Pablo Martínez-Camblor, Guillermo M Albaiceta

**Affiliations:** 1 Servicio de Hematología y Hemoterapia, Hospital Universitario Central de Asturias, Celestino Villamil s/n., 33006 Oviedo, Spain; 2 Departamento de Medicina, Universidad de Oviedo, Oviedo, Spain; 3 Servicio de Medicina Intensiva, Hospital Universitario La Fe, Valencia, Spain; 4 Servicio de Hematología, Hospital Universitario La Fe, Valencia, Spain; 5 Servicio de Medicina Intensiva, Hospital Son Llatzer, Palma de Mallorca, Spain; 6 Servicio de Hematología, Hospital Son Llatzer, Palma de Mallorca, Spain; 7 Servicio de Medicina Intensiva, Hospital Universitario Son Espases, Palma de Mallorca, Spain; 8 Servicio de Medicina Intensiva, Hospital de Navarra, Pamplona, Spain; 9 Servicio de Medicina Intensiva, Hospital Germans Trias i Pujol, Badalona, Spain; 10 Servicio de Medicina Intensiva, Hospital Universitario Miguel Servet, Zaragoza, Spain; 11 Oficina de Investigación Biosanitaria-FICYT, Oviedo, Spain; 12 Servicio de Medicina Intensiva, Hospital Universitario Central de Asturias, Oviedo, Spain; 13 CIBER-Enfermedades respiratorias, Instituto de Salud Carlos III, Madrid, Spain; 14 Departamento de Biología Funcional, Área de Fisiología, IUOPA, Universidad de Oviedo, Oviedo, Spain

## Abstract

**Introduction:**

Although the survival rates of hematological patients admitted to the ICU are improving, little is known about the long-term outcome. Our objective was to identify factors related to long-term outcome in hematological patients after ICU discharge.

**Methods:**

A prospective, observational study was carried out in seven centers in Spain. From an initial sample of 161 hematological patients admitted to one of the participating ICUs during the study period, 62 were discharged alive and followed for a median time of 23 (1 to 54) months. Univariate and multivariate analysis were performed to identify the factors related to long term-survival. Finally, variables that influence the continuation of the scheduled therapy for the hematological disease were studied.

**Results:**

Mortality after ICU discharge was 61%, with a median survival of 18 (1 to 54) months. In the multivariate analysis, an Eastern Cooperative Oncology Group score (ECOG) >2 at ICU discharge (Hazard ratio 11.15 (4.626 to 26.872)), relapse of the hematological disease (Hazard ratio 9.738 (3.804 to 24.93)) and discontinuation of the planned treatment for the hematological disease (Hazard ratio 4.349 (1.286 to 14.705)) were independently related to mortality. Absence of stem cell transplantation, high ECOG and high Acute Physiology and Chronic Health Evaluation II (APACHE II) scores decreased the probability of receiving the planned therapy for the hematological malignancy.

**Conclusions:**

Both ICU care and post-ICU management determine the long-term outcome of hematological patients who are discharged alive from the ICU.

## Introduction

Hematological patients admitted into an ICU experience high mortality rates. In response to this clinical problem, research has helped to identify prognostic factors related to intra-ICU and intra-hospital mortality. The result has been an improvement in the outcome over the last decade [1] as a consequence of a number of factors, including implementation of non-invasive mechanical ventilation [2], earlier intervention in septic shock [3] and better management of specific complications (such as tumor lysis syndrome) [4]. As most of the research has focused on intra-ICU or intra-hospital mortality, information regarding survival is limited to this period. More recently, some groups have extended the follow-up period to 6 to 12 months after the patients have been discharged from the ICU [5-10], but data regarding long-term survival (more than one year after ICU discharge) are scarce. This raises a concern about the validity of the classical predictive factors for intra-ICU or in-hospital mortality in predicting long-term survival. For instance, the need for mechanical ventilation is a well-known risk factor for death in the ICU, but its impact in long-term survival is largely unknown.

Moreover, there is increasing concern about the general condition of the patients at ICU discharge, including nutritional, neuromuscular and cognitive status. These factors, which are amenable to intervention and may impact the long-term survival and quality of life in unselected ICU patients, would be also relevant in a fragile population such as those with hematological malignancies. In addition, factors that determine the feasibility of subsequent chemotherapy cycles after ICU discharge and their impact in the long-term control of neoplastic disease have not been studied.

Here we report on long-term outcome in a population of hematological patients who survived ICU admission, and analyze the clinical factors influencing survival. We hypothesized that both intra-ICU and post-ICU variables could determine the suitability of a patient for receiving additional therapies, therefore determining the long-term outcome of this population. To test this hypothesis, we studied classical prognostic factors (such as mechanical ventilation, organ failure and neutropenia), as well as those related to the underlying disease and its evolution. In particular, we have analyzed the applicability of chemotherapy after ICU discharge and its relevance to hematological relapse and survival.

## Methods

**Figure 1:**
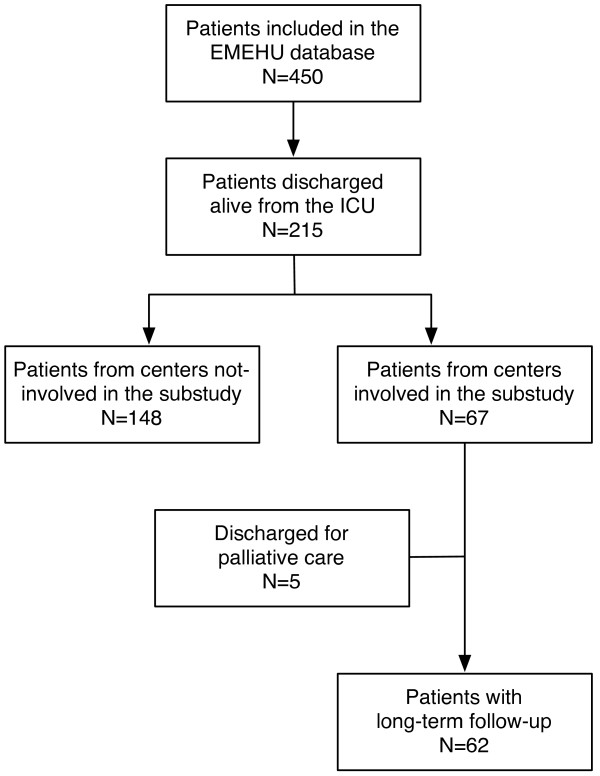
Patients included in the EMEHU study and subpopulation included in the present study.

The *Estudio Multicéntrico del Enfermo Hematológico en UCI* (EMEHU) study included all the hematological patients (including those with neoplastic or non-neoplastic disease), admitted due to medical or surgical reasons to one of 34 ICUs in Spain during the June 2007 to September 2008 period [11]. From the 450 patients included in the EMEHU database, 215 were discharged alive. Among these, 67 patients were discharged from one of the seven units participating in this sub-study. Patients discharged for palliative care were not included in the study. This resulted in a final sample of 62 ICU survivors, which constitutes the object of this investigation (Figure 1). To exclude selection bias a comparison was made between the patients included in the study and those from the non-participating centers. There were no differences in age, gender, hematological diagnosis, staging of the disease, percentage of transplanted patients, acute physiology and chronic health evaluation (APACHE)-II score at ICU admission or mortality (data not shown).

The study was approved by local ethics committees (see Acknowledgements) and written informed consent was obtained for each participant.

### Data collection

The EMEHU database included data on demographics, hematological disease, diagnosis at ICU admission, severity scores, treatments, infections and complications during the ICU stay. Neutropenia was defined as an absolute neutrophil count below 0.5 × 10^9^ L^-1^[12]. During the follow-up period after ICU discharge, data on the Eastern Cooperative Oncology Group (ECOG) score [13], relapse and treatment for the hematological disease were collected. According to the compliance with the pre-planned treatment for each hematological disease, four groups of patients were predefined: 1) those who did not require additional treatment (that is, patients in complete remission or transplanted patients without planned maintenance treatment); 2) patients who required and received full-dose treatment; 3) patients who required treatment and received it with modifications (reduced doses or delays); and 4) Patients who required treatment but did not receive it: for these patients the reasons for the changes in the therapeutic plan were recorded.

### Statistical analysis

Normality of all variables was studied using the Kolgomorov-Smirnov test. Data are expressed as mean ± SD or median (range) for variables with or without a normal distribution respectively. Univariate comparisons between survivors and non-survivors after ICU discharge were performed using the Student *t*-test, Mann–Whitney *U*-test or chi-square test. Those variables with a *P*-value lower than 0.05 for the difference were included in the multivariate survival analysis. In order to deal with time-dependent variables (that is, relapse), we used an extended Cox regression model. Hazard ratios (HR) with 95% confidence intervals were computed. A *P*-value lower than 0.05 was considered significant in this multivariate analysis. To evaluate the accuracy of the model over time, area under the incident/dynamic receiver operating characteristic (ROC) curve (AUC) was computed [14].

The four groups of patients resulting from the previously described classification based on the compliance with the pre-planned treatment were compared using one-way analysis of variance (ANOVA), Kruskal-Wallis ANOVA or chi-square test, as appropriate. In order to look for optimal classification criteria, significant variables were included in a decision-tree analysis. In particular, the chi-square automated interaction decision (CHAID) algorithm was used, and variables with a *P*-value lower than 0.05 were considered significant.

## Results

### General characteristics of the sample

During the study period, the seven participating hospitals admitted 161 patients. Sixty-two patients (39%) were discharged alive from the ICU and were analyzed. There were 36 male (59%) and 26 female (41%) patients. Age was 53 ± 16 (mean ± SD) years. The most frequent underlying disease was acute leukemia (38% of the patients) followed by lymphoma (24%). Most of the patients with acute leukemia were receiving remission-induction chemotherapy prior to ICU admission. Patients were transferred to the ICU from the emergency department in 18 cases (29%) or from the hospital ward in 44 cases (71%). The most common cause of ICU admission was acute respiratory failure (44% of the patients) followed by sepsis (35%) or shock (27%). There was only one surgical patient (with laparotomy due to perforated typhlitis), all the others being admitted due to medical reasons. All these characteristics of the study population are summarized in Table 1.

**Table 1:** Characteristics of the study population

	**Number of patients (%) or mean ± SD**
Comorbidities	
Coronary disease	5 (8)
Arterial hypertension	18 (29)
Diabetes	18 (29)
COPD	5 (8)
AIDS	2 (3)
Hepatic disease	3 (5)
Chronic renal failure	3 (5)
Non-hematologic cancer	4 (6)
Smoking	11 (18)
Tuberculosis	1 (2)
Hematological diagnosis	
Acute myeloid leukemia/ myelodysplastic syndrome	23 (38)
Acute lymphoid leukemia	4 (6)
Chronic myeloproliferative neoplasms	3 (5)
Chronic lymphocytic leukemia	5 (8)
Multiple myeloma	7 (11)
Hodgkin lymphoma	4 (6)
Non-Hodgkin lymphoma	11 (18)
Other	5 (8)
Timing of hematological diagnosis	
Previous to hospital admission	39 (63)
During the current hospital admission	18 (29)
In ICU	5 (8)
Stage of disease at ICU admission^a^	
Remission-induction	26 (44)
Remission	19 (30)
Relapse	16 (26)
Stem cell transplant	11 (17)
APACHE II score (mean ± SD)	22 ± 7
Diagnosis at ICU admission	
Acute respiratory failure	27 (44)
Sepsis	22 (35)
Cardiac failure	3 (5)
Cardiac arrest	1 (2)
Shock	16 (27)
Coma	2 (3)
Miscellaneous	6 (9)

^a^One patient with paroxysmal nocturnal hemoglobinuria cannot be included in this classification. COPD, chronic obstructive pulmonary disease; APACHE, acute physiology and chronic health evaluation.

**Figure 2:**
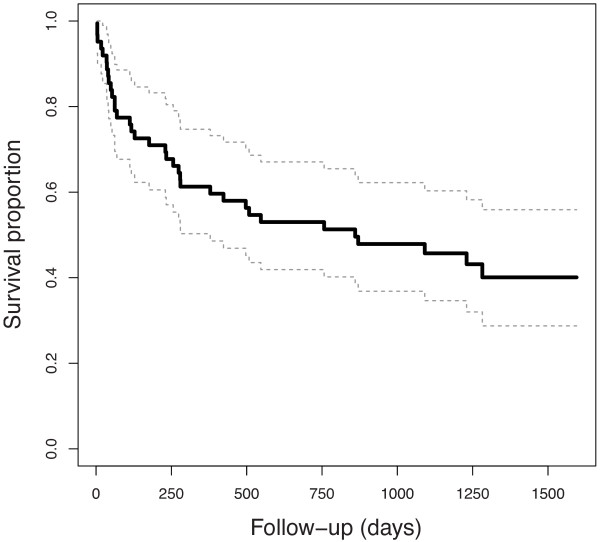
Overall survival of the whole population after ICU discharge (continuous black line) with 95% confidence intervals (dotted gray lines).

Patients were followed for a median time of 23 months (range 1 to 54). Thirty-seven patients (59%) died after ICU discharge, with a median overall survival of 18 months (range 1 to 54). Median follow-up time for survivors was 43 months (11 to 54). Figure 2 shows the survival curve for the study population.

### Risk factors for mortality after ICU discharge

First, a univariate analysis comparing survivors and non-survivors after ICU discharge was performed (Table 2). There were no differences in age or sex between these groups. Among the comorbid conditions, only a diagnosis of arterial hypertension was related to mortality. Regarding the hematological disease, there were no significant differences in mortality rates according to the main diagnosis, although there was a non-significant trend towards lower mortality rates in patients with lymphoma (HR 0.355, 95% CI 0.125, 1.006). Similarly, staging of the disease, referral site of the patient (emergency room or hospital ward) or history of stem-cell transplantation were not related to survival. Presence of neutropenia before or during the ICU stay was associated with a poor outcome. In fact, both variables were closely correlated. Therefore, we only considered neutropenia during the ICU stay (HR 2,024, 95% CI 1.042, 3.937) for inclusion in the multivariate analysis.

**Table 2:** Results of the univariate analysis studying the differences in variables collected before ICU discharge between long-term survivors and non-survivors

	**Alive**	**Dead**	***P*-value**
	**N = 26**	**N = 36**	
Age, years, mean ± SD	51 ± 14	55 ± 18	0.27
Gender, male/female	15/11	21/15	0.96
Hematological diagnosis			0.08
Acute leukemia/MDS	10	17	
cMPN	0	3	
CLL	2	3	
Multiple myeloma	2	5	
Lymphoma	11	4	
Other	1	4	
Phase of underlying disease^a^			0.95
Remission-induction	11	15	
Remission	8	11	
Relapse	6	10	
Arterial hypertension			0.04
Yes	4	14	
No	22	22	
Stem cell transplant	5	6	0.75
Neutropenia			
Intra-ICU	12	28	0.02
Pre-ICU	8	22	0.03
Thrombopenia previous to ICU	15	28	0.09
Diagnosis at ICU admission:			
Sepsis	10	12	0.67
Cardiac failure/infarction	0	3	0.26
Respiratory failure	13	15	0.69
Shock	4	11	0.23
Miscellaneous	6	2	0.06
APACHE- II score, mean ± SD	20.6 ±7.2	22 ± 6.7	0.42
Length of MV, days, median (range)	1 (0 to 42)	1 (0 to 62)	0.75
Length of NIMV, days, median (range)	0 (0 to 12)	0 (0 to 12)	0.60
Length of pre-ICU, days, median (range)	5 (0 to 66)	3 (0 to 26)	0.43
Length of ICU stay, days, median (range)	5 (1 to 58)	7 (1 to 68)	0.54
ECOG score at ICU discharge			0.001
0 to 2	25	22	
3 to 4	1	14	

Data are shown as number, mean ± standard deviation (SD) or median (range) for normally and non-normally distributed variables, respectively. MDS, myelodysplastic syndrome; cMPN, chronic myeloproliferative neoplasms; CLL, chronic lymphocytic leukemia; APACHE, acute physiology and chronic health evaluation; MV, mechanical ventilation; NIMV, non-invasive mechanical ventilation; ECOG, Eastern Cooperative Oncology Group. ^a^One patient with paroxysmal nocturnal hemoglobinuria cannot be included in this classification.

Among the data collected during the ICU stay, there were no differences between survivors and non-survivors in causes of admission, APACHE-II score, days of mechanical ventilation (either invasive or non-invasive) or complications during this period (data not shown). However, a high ECOG score at ICU discharge was significantly related to mortality (HR 7.28, 95% CI 3.572 to 14.850, for ECOG scores 3 to 4).

Finally, variables collected after ICU discharge were analyzed. There were significant differences in the relapse of the hematological disease (2 survivors versus 14 non-survivors respectively, chi-square test, *P* = 0.007). Compliance with treatment for the hematological disease also yielded statistically significant differences between survivors and non-survivors: Only 5 out of 26 survivors did not follow the previously planned therapeutic schedule, in contrast to 18 out of 36 among non-survivors (chi-square test, *P* = 0.02).

**Figure 3:**
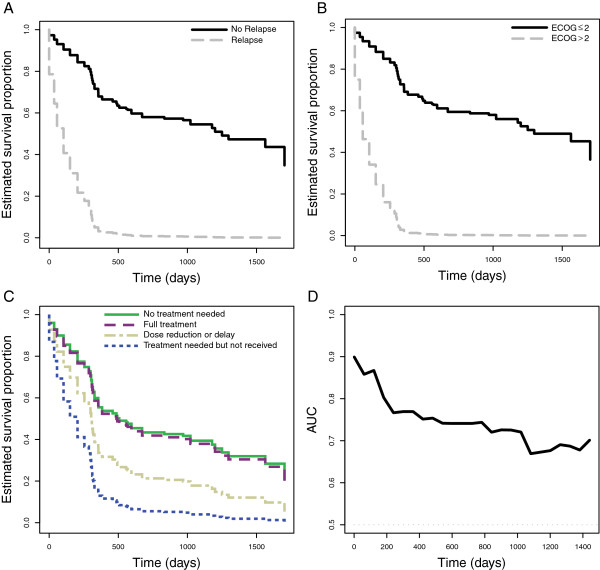
**Survival curves.** Estimated survival curves according to the Cox regression model showing the impact of relapse **(A)**, Eastern Cooperative Oncology Group (ECOG) score **(B)** and each therapeutic group (no need for further treatments, treatment received without changes or delays, treatment received with dose reductions or delays, treatment needed but not received) **(C)**. The accuracy of the regression model, measured as the area under the receiver operating characteristic curve (AUC) over time is shown in panel **D**.

The five variables with a *P*-value lower than 0.05 were included in a multivariate model (Table 3 and Figure 3). In this analysis, relapse (Figure 3A), ECOG score at ICU discharge (Figure 3B) and compliance with the scheduled treatment for the hematological disease (Figure 3C) were significantly related to post-ICU mortality. The accuracy of this model was evaluated over time by estimation of the AUC (Figure 3D). As shown, accuracy was good immediately after ICU discharge (AUC 0.90) and decreased to 0.77, 0,74 and 0.72 after one, two and three years, respectively.

**Table 3:** Multivariate analysis for survival

	**Hazard ratio**	**95% Confidence interval**
ECOG score > 2 at ICU discharge	11.150	4.626, 26.872
Relapse after ICU discharge	9.738	3.804, 24.93
Compliance with therapy		
Finished treatment pre-ICU	1	
Full treatment	1.075	0.319, 3.622
Dose reduction or delay	2.172	0.629, 7.501
No treatment	4.349	1.286, 14.705

ECOG, Eastern Cooperative Oncology Group.

### Therapeutic strategies after ICU discharge

Considering the impact of post-ICU treatment on survival we analyzed factors that determined the probability of patients to receive pre-planned treatment for hematological disease. Among the 62 studied patients, 15 did not need further treatment, 24 patients completed the pre-planned treatment after ICU discharge, 12 patients received treatment with dose reductions or delays (4 due to infection and the remainder due to a medical decision) and 11 patients did not receive treatment for their hematological disease (all due to a medical decision).

**Figure 4:**
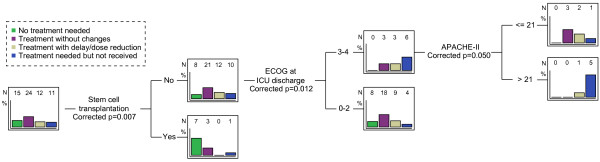
**Decision-tree analysis showing the variables that significantly discriminated among the different therapeutic groups.** APACHE-II, acute physiology and chronic health evaluation score; ECOG, Eastern Cooperative Oncology Group score.

The differences among these groups were studied using a decision-tree analysis. The three variables that yielded significant differences were stem-cell transplant, ECOG score at ICU discharge and APACHE-II score (Figure 4). As expected, most transplanted patients did not require more treatment after ICU discharge. However, patients with ECOG score >2 at ICU discharge, and specifically those with an APACHE-II score above 21, received treatment with dose reductions or delays, or discontinued the treatment more frequently.

## Discussion

In this work we have analyzed the evolution of a cohort of hematological patients after an ICU stay in order to identify the factors related to long-term survival after ICU discharge. Although the sample size of our study could result in low statistical power, the robust statistical analysis used, including univariate and multivariate analysis, and restrictive cut-off points, have helped to identify some relevant variables related to long-term outcome. In this sense, an ECOG score >2 at ICU discharge, the relapse of the hematological neoplasm and the absence of compliance with the scheduled therapy for the underlying disease after ICU discharge predicted worse survival in this cohort. However, it must be recognized that other variables may have not been detected due to the sample size. In this sense, larger studies will be required to validate our findings.

The need for data on long-term survival of hematological patients after ICU discharge has been recently highlighted due to their increased acceptance in ICUs [15], improvements in survival [1] and awareness of resource consumption [16,17]. Our work presents data after a median follow-up time of 23 months, with 59% mortality and a median survival of 18 months.

As most of the previously published studies dealing with critically ill hematological patients are limited to a follow-up period of 6 to 12 months [5-9,18], our results are difficult to compare. In spite of this limitation, high mortality rates after ICU discharge have been uniformly described. This high number of deaths must be added to the high ICU mortality of this population (about 40 to 50% in the published literature [5-8,10] and 61% in our series). Nonetheless, these findings were expected, as both an ICU stay [19] and a diagnosis of a hematological neoplasm are risk factors for death in the general population. Moreover, the most prevalent hematological diagnosis in our cohort was acute leukemia during the induction phase, a condition with high risk of infections and death [20]. Due to the diagnostic heterogeneity of our sample we cannot properly assess how the ICU stay modifies the course of these diseases. With this limitation in mind, we can compare our survival rates with an unselected population of patients with acute myeloid leukemia [21]. Our data show one-year mortality higher than the 10 to 15% reported for the unselected population. However, long-term survival in our study was about 40%, which is not very different from the 50% reported for the whole cohort of patients with leukemia. The impact of an ICU stay in the course of specific diseases should be addressed in future studies.

The three predictive factors for survival in our study were ECOG score at ICU discharge, relapse of hematological disease and adherence to the planned therapy after discharge. The ECOG score is a simple and easily applicable test that reflects the patient's response to the tumor and quantifies the performance status of the subject. It has demonstrated a prognostic value in virtually all hematological neoplasms, independently of other clinical and biological variables [22-25]. In the context of this study, it is reasonable to think that the consequences of intensive therapies required during the ICU stay may have impact on the final ECOG score at ICU discharge. Recently in a large cohort, poor performance status has been demonstrated to be related to an increased risk of intra-hospital death [10]. The second prognostic factor for survival is relapse of underlying disease. This is not a surprising result, considering that relapse confers a kind of resistance to chemotherapy that is exceptionally difficult to overcome except with aggressive therapies, like stem-cell transplantation [26]. Finally, discontinuation of the scheduled treatment for the hematological disease is the third factor related to a poor outcome. This risk is partially avoided when treatment is given, even with dose reductions or delays.

Due to the relevance of this last factor, which has not been explored previously, we focused on the variables that could predict the adherence to the subsequent hematological treatment. The decision-tree analysis allows us to identify the most relevant parameters that determine allocation to one group. The first variable that determined the probability of treatment continuation was stem-cell transplantation. As expected, patients after stem-cell transplantation or those in complete remission do not need further treatment for their disease, and show a good prognosis after ICU discharge. Conversely, those in the remission-induction phase or those with relapse usually need additional treatments. It must be noted that up to 50% of the patients in the remission-induction phase do not receive full treatment after ICU discharge. In these patients, the ECOG score appears again as a critical factor that discriminates among groups with different probabilities of receiving a complete chemotherapy treatment. According to our previous discussion, patients with an ECOG score >2 at ICU discharge have a substantially decreased functional reserve that hampers their capacity to tolerate an aggressive treatment, such as chemotherapy. This finding highlights the critical importance of strategies aimed at minimizing the negative impact of the ICU stay and preserving the patient's status [27]. Finally, the APACHE-II allows further discrimination among the patients with high ECOG scores. A high APACHE-II score decreased the probability of continuation of therapy once the patient has been discharged from the ICU. It has been demonstrated that early ICU admission improves the outcome in this population [10,28]. The admission before the development of multiple organ dysfunction could be related to lower APACHE-II scores and a better compliance with the treatment after ICU discharge, thus improving the long-term outcome. However, our data do not allow identification of other factors that may have special relevance, such as specific organ failure responsible for the medical reasons behind the changes in the therapeutic plan.

Collectively our study suggests that management of critical hematological patients goes beyond the ICU and represents a challenge for both hematologists and intensivists: once a hematological patient is admitted to the ICU, physicians should consider the need to follow the therapeutic plan and not only the immediate risk of death. Previous studies have demonstrated that when needed, continuation of the chemotherapeutic regimen after ICU admission could improve the outcome of these patients [29]. Our results extend this observation, and suggest that an active therapeutic strategy must be taken after ICU discharge. If this goal cannot be accomplished, the ICU stay could be an exercise of futility due to the high risk of death after discharge. These findings should be taken into account when considering readmission of one of these patients, due to the poor outcome of those discharged alive but unable to continue their treatment.

## Conclusions

Our results show that outcome of hematological patients who are discharged alive from the ICU depends on their functional status and the adherence to the planned therapy for their disease. Moreover, these two factors are closely related. These findings illustrate the relevance of strategies to reduce the consequences of an ICU stay and add new parameters to consider in the management of this fragile population.

## Key messages

• Performance status, relapse of the hematological disease and continuation of the scheduled treatment for the hematological disease are the key factors that determine long-term survival of hematological patients after an ICU stay.

• The ability to continue the planned treatment for the hematological disease depends on performance status and APACHE-II score. Patients who have completed the stem-cell transplant procedure or do not need more treatment have a good outcome.

## Abbreviations

ANOVA: Analysis of variance; APACHE: Acute physiology and chronic health evaluation; AUC: Area under the curve; CLL: Chronic lymphoid leukemia; cMPN: Chronic myeloproliferative neoplasm; COPD: Chronic obstructive pulmonary disease; ECOG score: Eastern Cooperative Oncology Group score; HR: Hazard ratio; MDS: Myelodysplastic syndrome; MV: Mechanical ventilation; NIMV: Non-invasive mechanical ventilation; ROC: Receiver operating characteristic.

## Competing interests

The authors declare that they have no competing interests.

## Authors’ contributions

TB and GMA designed the study. TB, EVP, JBo, IJ, MB, JBa, JIA, JI, PM and VGS collected the data. TB, PMC and GMA performed the analysis. All the authors discussed the results. TB and GMA wrote and reviewed the article. All the authors read and approved the manuscript.
